# Specular Reflection Suppression through the Adjustment of Linear Polarization for Tumor Diagnosis Using Fluorescein Sodium

**DOI:** 10.3390/s22176651

**Published:** 2022-09-02

**Authors:** Sangyun Lee, Kicheol Yoon, Jungmin Kim, Kwang Gi Kim

**Affiliations:** 1 Department of Health and Safety Convergence Sciences, Korea University, 145, Anam-ro, Seongbuk-gu, Seoul 02841, Korea; 2 Department of Health and Environmental Convergence Sciences, Korea University, 145, Anam-ro, Seongbuk-gu, Seoul 02841, Korea; 3 Medical Devices R&D Center, Gachon University Gil Medical Center, 21, 774 Beon-gil, Namdong-daero, Namdong-gu, Incheon 21565, Korea; 4 Department of Biomedical Engineering, College of Medicine, Gachon University, 38-13, 3 Beon-gil, Dokjom-ro 3, Namdong-gu, Incheon 21565, Korea; 5 Department of Biomedical Engineering, College of Health Science, Gachon University, 191 Hambak-moero, Yeonsu-gu, Incheon 21936, Korea; 6 Department of Health Sciences and Technology, Gachon Advanced Institute for Health Sciences and Technology (GAIHST), Gachon University, 38-13, 3 Beon-gil, Dokjom-ro 3, Namdong-gu, Incheon 21565, Korea

**Keywords:** fluorescein sodium, fluorescence imaging device, tumor diagnosis, specular reflection, linear polarizing filter

## Abstract

In tumor surgery, the edges of the tumor can be visually observed using a fluorescent contrast agent and a fluorescent imaging device. By distinguishing it from normal tissues and blood vessels, it is possible to objectively judge the extent of resection while visually observing it during surgery, and it guarantees safe tumor resection based on more information. However, the main problem of such an imaging device is the specular reflection phenomenon. If specular reflection overlaps with important lesion locations, they are a major factor leading to diagnostic errors. Here, we propose a method to reduce specular reflection that occurs during tumor diagnosis using a linear polarization filter and fluorescent contrast agent. To confirm the effect of removing specular reflection, a self-made fluorescein sodium vial phantom was used, and the reliability of the results was increased using a large animal (pig) test. As a result of the experiment, it was possible to obtain an image in which specular reflection was removed by controlling the rotation angle of the filter by 90° and 270°, and the same results were confirmed in the phantom experiment and the animal experiment.

## 1. Introduction

The lesion classification and observation method using fluorescein (FL) sodium (yellow dye) is widely used in ophthalmology, dentistry, and oncology surgery fields. It is used in ophthalmology to identify inflammation of the eyeball. In dentistry, it is widely used to observe periodontal disease and inflammation. In the field of oncology surgery, it is widely used to distinguish the removal status of tumors or the location of tumors [1,2,3]. During oncology surgery, the extent of resection of the tumor must be determined before surgery. It is crucial to remove the tumor to the maximum extent possible and minimize the damage to surrounding normal tissues and blood vessels. This is a method of increasing the 5-year survival rate of patients and preventing recurrence [4]. Therefore, the main aim of surgical operation is to excise the tumor safely and completely. However, the tumor is mixed with blood vessels and has the same color and shape. Therefore, it is difficult to distinguish and observe the tumor margin with the naked eye. This hinders the complete resection of the tumor by separating the tumor from the vascular boundary during surgical operation [5,6]. Therefore, to overcome these limitations, a fluorescence imaging device is used [7,8,9,10,11]. This device can distinguish between tumors and blood vessels using a fluorescence contrast agent. This method enables one to visually check the boundary between the tumor and blood vessels. In addition, it can objectively determine the extent of resection during a surgical operation. Therefore, the tumor can be resected as safely as possible based on more information [12,13]. Therefore, there is a method for discriminating tumors and normal tissues through various fluorescence staining techniques using an imaging device. Representatively, the use of rhodamine fluorescence allows the selective monitoring of exogenous and endogenous lysosomal ONPP- in vivo using fluorescence emission properties at 733 nm [14]. BODIPY series has attracted considerable attention due to its excellent photophysical properties such as large molar extinction coefficient, high-fluorescence quantum yield, adequate redox potential, and good safety. In particular, it is being used as a fluorescent sensor, showing high sensitivity and selectivity to Hg (II) ions [15,16]. In addition, various synthetic methods available for the preparation of BODIPY dyes allow the incorporation of modifiable/efficient fluorescent groups. Various techniques have been developed to prepare novel fluorescent dyes due to their excellent electronic and spectral properties [17]. The use of the fluorescent material utilizes the secretion of specific proteins or peptides from tumor cells into the bloodstream. When proteins made specifically in tumor cells are degraded by various enzymes and released into the bloodstream, we can determine the pattern of proteins or peptides secreted through the blood. When a protein is analyzed by liquid chromatography/mass spectrometry (LC-MS), the relative amount of the target protein can be known, and through this, a specific pattern in cancer that is different from normal can be identified [18].

However, the fluorescence imaging apparatus has a disadvantage in that specular reflection occurs in the captured image due to the light emitting diode (LED) light used in such devices. The specular reflection by the LED overlaps with the lesion location or obstructs the observation field. Therefore, the occurrence of specular reflection prevents accurate lesion observation. This is the main cause leading to diagnostic errors [13]. To address these problems, research methods for specular reflection suppression have been reported, but research cases are still lacking [13,19].

In the medical field, most approaches have attempted to minimize specular reflection by adjusting the direction angle of the camera. There are two methods of capturing the polarization effect of the camera: a method of irradiating the focal point of the LED in various directions and a method of controlling the focal length of the LED beam [20]; however, adjusting the direction angle and focal length of the LED beam is not easy [21]. In addition, the best tool for specular reflection suppression is a method of detecting specular reflection. These specular reflections are generated in the process of spatial transformation with red–green–blue (RGB) color functions for the brightness and color of the camera [22]. The detected specular reflection removes the reflection region through deconvolution analysis in the wavelength band. The color image lost due to specular reflection removal uses a separate function to restore the lost color [22]. This reflected wave removal process requires extensive data, calculations, and complex mathematical operations [22]. The method of collecting frames through continuous shooting requires the reference image to be trained in advance [23]. This method compares the interval between the actual shooting frames and the reference training image interval and calculates the sum and difference. Therefore, by removing the image for the calculated difference, the calculated value can be used to remove the reflected light [23]. However, this method requires a complex collection process for lesion data, normal tissue data, and data for learning reference images [24].

Another method is to use a polarizing filter in the camera. In addition, there is a method that uses a sensor to automatically detect changes in the image, and it detects only the RGB values of the image [25,26,27,28]. However, the method of applying the polarizing filter reduces the shooting radius owing to material loss and provides a dark image. The RGB value detection method does not consider image loss and distortion due to material loss in the filter. Methods for suppressing specular reflection of photographed data require research to overcome the complex process of data collection and mathematical operation [25,26,27,28]. Another method of reducing specular reflection is a gradient image transmission method. In this way, the captured image may be set to have high pixels and specular reflection may be set to have low pixels. Therefore, the above method performs gradient mapping through pixel optimization [29]. A similar mapping method collects the high-pixel data for images and low-pixel data for specular reflection and subsequently learns these data [30,31]. This method induces gradient mapping such that only high pixels are selected. The selected pixels provide images with effectively reduced specular reflection [30,31]. This method requires complex analysis and algorithm design [29].

This paper proposes a method for removing specular reflection in a fluorescence imaging device. To remove specular reflection, the linear polarization filter controls vertical and horizontal polarization through rotation. The polarized wave is adjusted to horizontal polarization through rotation control, and the rotation angle of the filter for horizontal polarization control becomes 90°. Therefore, the angle of vertical polarization and horizontal polarization feature a 90° difference.

## 2. Analysis of Fluorescence Emission and Specular Reflection Occurrence

**Figure 1:**
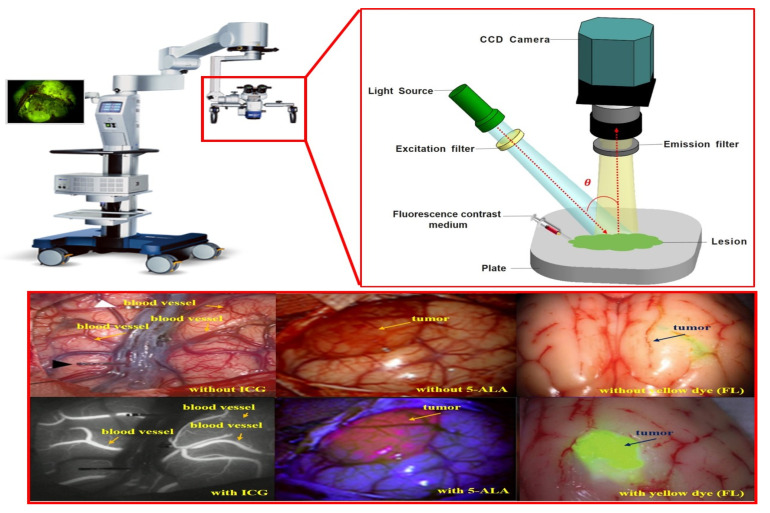
Fluorescence imaging module using fluorescence contrast agent.

As shown in Figure 1, the fluorescence imaging device acquires images through injection of a fluorescence contrast agent, irradiation with an external light source, and imaging. At this time, blood vessels or tumors are classified by color and observed through images. The purpose of using a fluorescence contrast agent is to observe status of blood vessel flow, remove the tumor through fluorescent staining, and follow up on the state of the tissue remaining after tumor resection [32,33,34].

Fluorescence imaging devices are classified into fixed, pen, and handheld types, as shown in Table 1 [29,35]. These instruments are used to observe fluorescence images during surgery.

**Figure 2:**
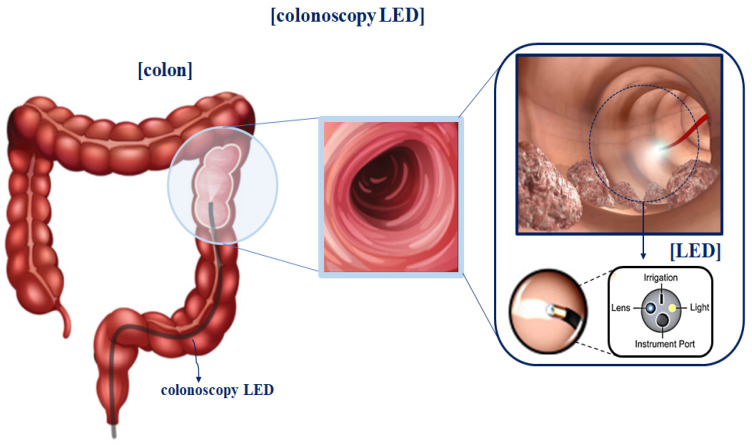
Camera structure of fluorescence imaging device.

As shown in Figure 2, the device’s structural features include a built-in LED light to brightly illuminate a dark field of view around the imaging camera [36]. The observation method using a fluorescence contrast agent secures the field of view to effectively observe the tissue from the operating microscope, while the LED light is brightly lit on the tumor [4,21].

**Figure 3:**
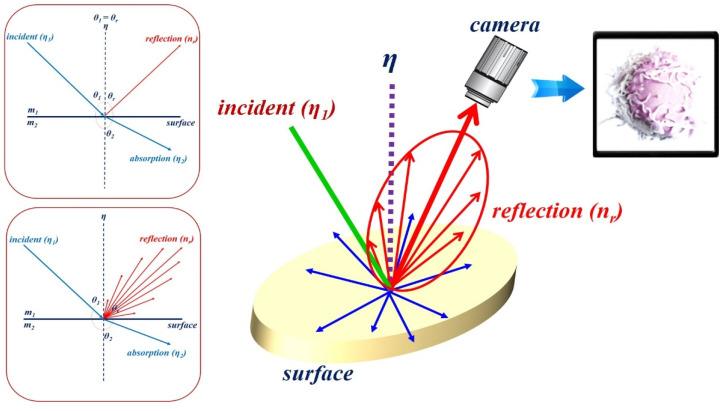
Illustration of Snell’s law.

However, specular reflection in observational images is attributed to the influence of tissue density and mucosal moisture status. As shown in Figure 3, for the light ($\eta_{1}$) irradiated from the LED, according to Snell’s law, a part of the light ($\eta_{2}$) has a refraction angle ($\theta_{2}$) that is absorbed by the tissue, and the remaining light is reflected ($\eta_{r}$). Therefore, *η*_1_ is equal to $\eta_{r}$ ($\eta_{1}$ = $\eta_{r}$ ($\theta_{1}$ = $\theta_{r}$)) [37]. As a result, as in Equation (1), a difference between the incident angle ($\eta_{1}$) and reflection angle ($\eta_{r}$) occurs, resulting in specular reflection.

(1) $$tan\theta_{2}=\frac{\eta_{1}\eta_{r}}{\eta_{2}}=tan^{-1}\theta_{r}$$

**Figure 4:**
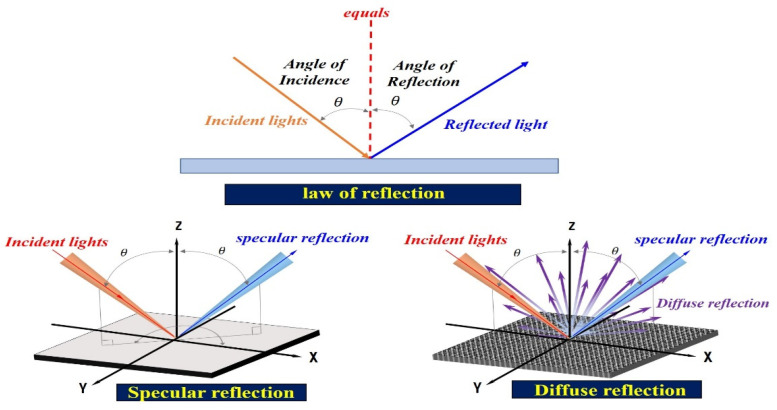
Diffused reflection resulting from the varying reflected specular ($\eta_{r}$).

At this time, the reflected light ($\eta_{r}$) is generated in the form of diffuse reflection ($\eta_{r}$), as shown in Figure 4. Depending on the angle of the irradiated light, it is reflected in various directions, and the reflected light is captured by the camera [37,38,39].

Thus, the specular reflection angle is changed from 0 to 180°, and it is called diffuse reflection ($\eta_{dif}$) [40]. The specular reflection ($\eta_{r}$) is given by Equation (2). Accordingly, $\lambda_{dif}$ includes $\theta_{1}$ and $\theta_{r}$, where $\lambda_{dif}$, $\theta_{1}$, and $\theta_{r}$ are the wavelength of diffuse reflection and the incident and reflection angles, respectively.

(2) $$\eta_{r}=\eta_{dif}\left(\lambda_{dif}\theta_{1}\theta_{r}\right)$$

**Figure 5:**
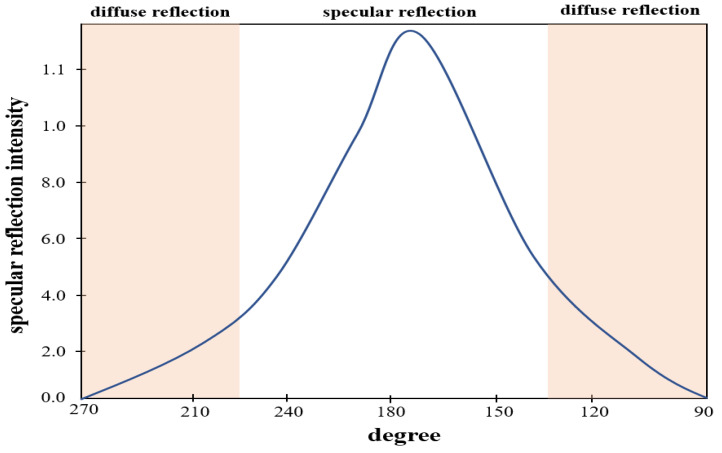
Distribution of light intensity according to the angle of light generation.

At this time, the intensity (*I_ηr_*) of the specular reflection ($\eta_{r}$) is strong, and the intensity (*I_ηdif_*) of the diffuse reflection ($\eta_{dif}$) is weak, and the light spreads from 0 to 180°, as shown in Figure 5.

**Figure 6:**
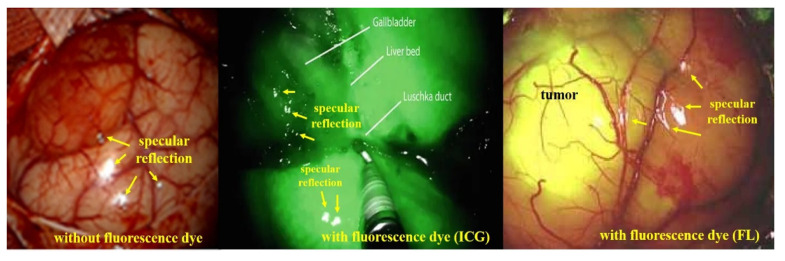
Comparison of specular reflection between indocyanine green (ICG) and fluorescein (FL) sodium in fluorescence imaging.

Therefore, as shown in Figure 6, images acquired based on specular reflection generate white light during endoscopy, tumor observation, and blood circulation [39]. If such specular reflection occurs in an important lesion area of the image, it may interfere with the observation field and cause a diagnosis error [34].

Therefore, a filter is used to remove such specular reflection. There are several types of filters used, such as ultraviolet (UV) filters, skylight filters, polarizer (PL) filters, and neutral density (ND) filters [41]. UV and skylight filters are mainly used to reduce specular reflection caused by UV rays [42]. The PL filter is used to reduce diffuse reflection, and the ND filter is used to reduce specular reflection by uniformly reducing the amount of light to balance the color image in its entirety [37]. However, these filters are mainly suitable for shooting in bright natural environments. The PL filter is widely used because it shows excellent performance in terms of reducing light reflection [42]. However, PL filters have the disadvantage of darkening the background; this was first studied in phantom experiments and published in subsequent papers [13]. Therefore, if a PL filter is used in a state that only relies on LED light in a dark human body, light reflection is effectively reduced. However, the method of using the PL filter has limitations in securing the lesion field of view owing to the dark image [13]. Therefore, research to effectively remove specular reflection in a dark environment and observe the lesion in a bright field of view is necessary.

## 3. Elimination of Specular Reflections

**Figure 7:**
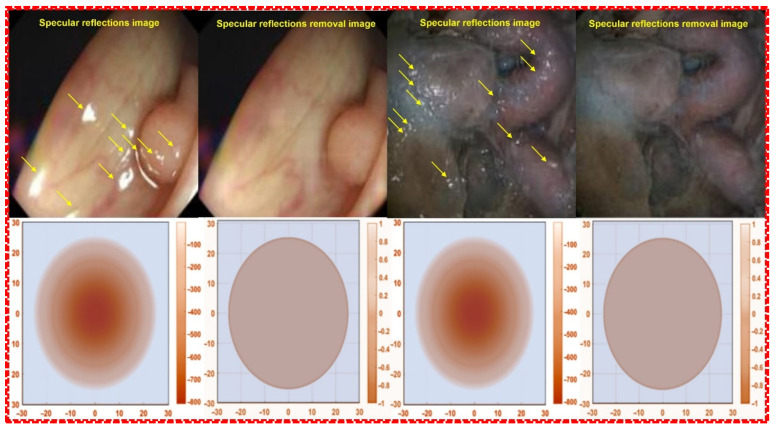
Images before and after the removal of specular reflection during colonoscopy.

The occurrence of specular reflections displayed in yellow in the microscopic image interferes with the accurate identification of surgical sites and lesions and is a factor limiting accurate surgery. To solve this problem, after analyzing the characteristics of polarization and Malus’ law for specular reflection simultaneously, the polarization direction of the incident light is controlled to remove the light previously observed as specular reflection, as shown in Figure 7, to obtain a clear image [39].

After the camera is photographed the region of interest, the energy corresponding to the image includes vertical polarization (V_in_) and horizontal polarization (H_in_) [25,29]. Accordingly, the linearly polarized light ($\bar{E}$) direction (*z*) is given by Equations (3) and (4) [43].

(3) $$E_{x}=xE_{ref}e^{j\left(\omega t-\theta_{1}z\right)}$$

(4) $$E_{y}=yE_{ref}e^{j\left(\omega t-\theta_{1}z\right)}$$

**Figure 8:**
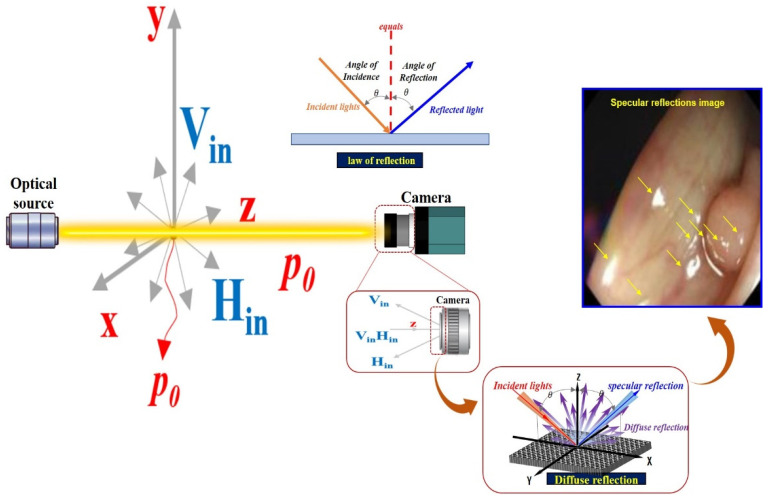
Specular reflection due to diffuse reflection after incidence with vertical polarization and vertical polarization camera.

If an image is acquired from a camera in an environment without a polarization filter, the images corresponding to the vertical (V_in_) and horizontal (H_in_) polarizations are incident on the camera, as shown in Figure 8 [44,45,46]. Thus, the linearly polarized light ($\bar{E}$) direction (*z*) corresponding to the imaging of the vertical polarization (V_in_) and horizontal polarization (H_in_) is expressed by Equation (5) [43].

(5) $$E_{x,\;y,z}=x\left[E_{ref,x}e^{j\left(\omega t-\theta_{1}z\right)}\right]+\left[E_{ref,y}e^{j\left(\omega t-\theta_{1}z\right)}\right]$$

**Figure 9:**
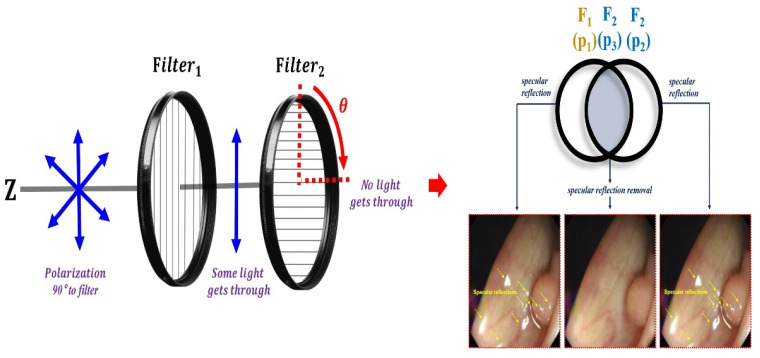
Adjusting the rotation angle using the filter and placing the filter.

Incident waves to V_in_ and H_in_ through camera shooting have the property of diffuse reflection. At this time, the property of diffuse reflection includes the size of *A*^2^, as shown in Equation (6).

(6) $$E_{x}+E_{y}=A^{2}cos^{2}\left(\omega t-\theta_{1}z\right)+A^{2}cos^{2}\left(\omega t-\theta_{1}z-\frac{\pi }{2}\right)\;=A^{2}cos^{2}\left(\omega t-\theta_{1}z\right)+A^{2}sin^{2}\left(\omega t-\theta_{1}z\right)\;=A^{2}\left[cos^{2}\left(\omega t-\theta_{1}z\right)+sin^{2}\left(\omega t-\theta_{1}z\right)\right]=A^{2}$$

where ω is the angular frequency, that is, 2πf(ω=2π). Accordingly, f is termed the frequency. Thus, $\lambda =\frac{c}{f}$ (c is the velocity of light: 3 × 10^8^ (m/s)) [47]. To suppress the diffuse reflection characteristics, the vertical polarization (V_in_) must be removed. Therefore, in the method for removing the vertical polarization wave (V_in_), the rotation angle should be controlled between the two types of the first filter (Filter_1_) and the second filter (Filter_2_), as shown in Figure 9. The first filter (Filter_1_) is fixed, and the second filter (Filter_2_) is rotated.

Thus, the filter can sufficiently remove the specular reflection. Therefore, the suppressed specular reflection ($E_{x,y,z}$) is expressed as Equation (7).

(7) $$E_{x,y,z}=E_{y}cos^{2}\left(\omega t-\theta_{1}z-\frac{\pi }{2}\right)\;\mathrm{at}\;A^{2}=0$$

**Figure 10:**
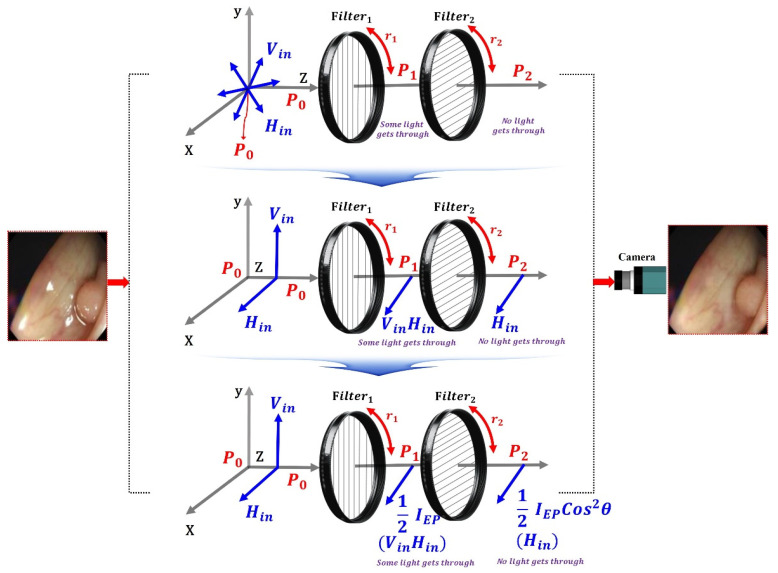
Specular reflection removal analysis through the adjustment of rotation angle with the polarizing filter.

Filter_1_ is fixed as shown in Figure 10, and when the rotation direction angle (r_1_) is 0°, V_in_ and H_in_ incident on the filter have vertical polarization and horizontal polarization at P_0_. At this time, when V_in_ and H_in_ pass through Filter_1_, both V_in_ and H_in_ are changed to the horizontally polarized waves at P_1_. Assuming that the direction angle (r_1_) of Filter_1_ becomes 0° and the direction angle (r_2_) of Filter_2_ rotates, V_in_ and H_in_ passing through the filter are polarized as in Equations (8)–(11), affecting P_3_. Therefore, the specular reflection can be sufficiently removed.

Therefore, when the specular reflection intensity (*I_ref_*) is rotated, the specular reflection intensity (*I_ref_*) with respect to the direction angle (r_2_) of the filter is approximately 3/4 (π/4) when the phase angle (*θ*) decreases to 60° [44].

(8) $$I_{ref}=I_{Ep}cos^{2}\theta \;$$

(9) $$2cos=2I_{Ep}cos^{2}\theta \;$$

(10) $$1/2I_{Ep}cos^{2}\theta \;$$

(11) cos90° 

For example, the intensity of specular reflection (*I_ref_*) is when the filter of Filter_1_ is 0° and the filter of Filter_2_ is 90°; the phase angle (*θ*) of the specular reflection intensity passing through the filter of Filter_2_ is the difference of 90° (*θ* = 90°).

Therefore, as shown in Table 2, the intensity of the current changes according to the rotation angle. As a result, V_in_ does not pass, and H_in_ passes. Therefore, the specular reflection intensity (*I_EP_*) for P_3_ is reduced by more than half compared with the specular reflection intensity *(I_ref_*) of P_2_, as shown in Table 3.

**Figure 11:**
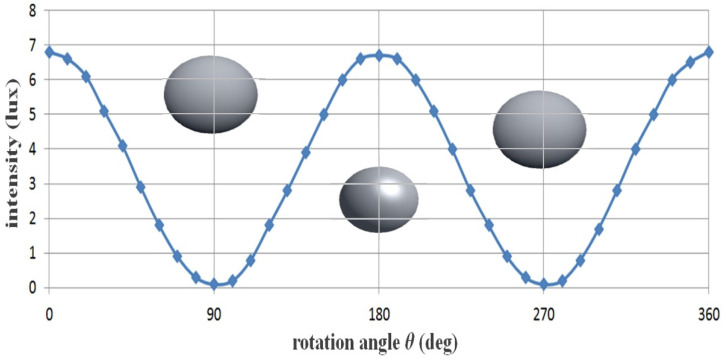
Changes in specular reflection intensity according to the rotation axis of the filter.

From the table, assuming that Filter_1_ is fixed at 0°, Filter_2_ is sequentially rotated (r_2_) from 0 to 360°, and the specular reflection intensity decreases and increases repeatedly, as shown in the sine wave form in Figure 10. This phenomenon will continue to repeat itself. Therefore, when the rotation angle (r_2_) of Filter_2_ is 90° or 270°, the intensity of specular reflection becomes 0 mW/cm^2^, and a phenomenon wherein specular reflection occurs is removed. Furthermore, when the rotation angle (r_2_) is 0°, light (specular reflection, *I_EP_*) will pass through the second polarization filter (Filter_2_), as shown in Figure 11, and thus, the intensity of specular reflection will have the maximum value. Overall, the intensities for *I_EP_* and *I_ref_* are the same, and the intensities have an equilibrium relationship with each other.

If the rotation axis (r_2_) of the second filter (Filter_2_) reaches 90°, *I_EP_* and *I_ref_* (Filter_1_ ≠ Filter_2_ = 90° difference of the filter) exhibit a cross relationship. Thus, *I_EP_* features a maximum value (*I_EP_* = 1), and *I_ref_* features a minimum value (*I_ref_* = 0). Therefore, the intensity of the specular reflection is diminished, and the image quality is increased. If the rotation angle (r_2_) of the filter deviates from 90° (<100°), *I_EP_* changes to the minimum value (*I_EP_* ≒ 0), and *I_ref_* changes to the maximum value (*I_ref_* ≒ 1). Finally, *I_EP_* and *I_ref_* feature the same value (*I_EP_* = *I_ref_*), and this phenomenon is repeated.

To summarize, the following is the method for maximally suppressing specular reflection: when the first filter (Filter_1_) is 0°, the second filter (Filter_2_) should be 90°. As a result, the polarized wave (p_3_) that has passed through Filter_2_ outputs only the polarized wave with reduced specular reflection (H_in_ = 0). The intensity of light loses its maximum value, and eventually, the intensity of the light starts to decrease gradually. When the rotation angle (r_2_) is 90° (270°), the intensity of the specular reflection (*I_ref_* = 0) becomes 0. If the rotation axis (r_2_) of the second filter (Filter_2_) reaches 90°, *I_EP_* and *I_ref_* (Filter_1_ ≠ Filter_2_ = 90° difference of the filter) exhibit a cross relationship. Thus, *I_EP_* features a maximum value (*I_EP_* = 1) and *I_ref_* features a minimum value (*I_ref_* = 0). Therefore, the intensity of the specular reflection is lost, and the image quality is increased. If the rotation angle (r_2_) of the filter deviates from 90⁰ (<100°), *I_EP_* changes to the minimum value (*I_EP_* ≒ 0), and *I_ref_* changes to the maximum value (*I_ref_* ≒ 1). Finally, *I_EP_* and *I_ref_* attain the same value (*I_EP_* = *I_ref_*), and this phenomenon is repeated.

## 4. Experiment Configuration

To realize the effect of removing specular reflection, this study employs two methods. The first method is an experiment using a fluorescein sodium substance vial. In this method, the specular reflection state is tested in a state in which one vial of fluorescein sodium is filled into a micro tube. The second method employs preclinical (animal testing) tests to obtain reliable results. The fluorescein sodium substance vial was manufactured in house, and the manufacturing method is introduced in Section 4.1. In addition, the preclinical trial preparation process is introduced in Section 4.2. Introducing the experimental results for specular reflection removal, Section 4.3 discusses the LED irradiation, camera shooting method, device configuration, and experimental preparation process.

### 4.1. Fluorescein Sodium Substance Vial Production

**Figure 12:**
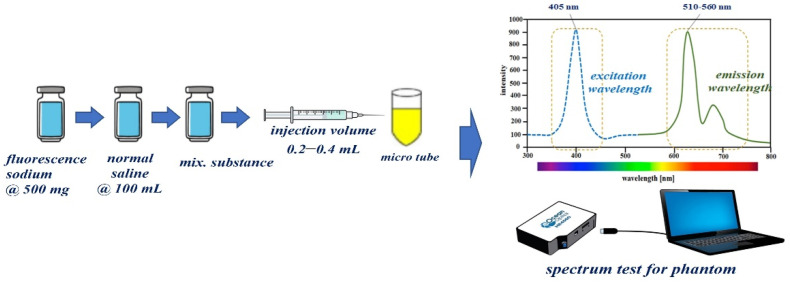
Proposed manufacturing method of one-vial phantom using fluorescein sodium.

To obtain the results for the specular reflection removal effect, we make our own vials capable of fluorescence expression using fluorescein sodium (yellow dye). For vial fabrication, the material is prepared by mixing fluorescein sodium and normal saline, as shown in Figure 12. For 5.0 mg/mL dilution from fluorescein sodium (500 mg) and normal saline (100 mL), 0.8 mL is injected into a micro tube (1 mL) to develop a one-vial drug-based phantom that can be compared with the phantom. For the phantom experiment, five vials were prepared for easy comparative evaluation. As for the experimental method, the five vials are photographed under the condition that specular reflection occurs before the filter is applied, and the remaining five vials are observed after the filter is applied, and the specular reflection removal state is observed.

### 4.2. Preclinical Trial Preparation Procedure

**Figure 13:**
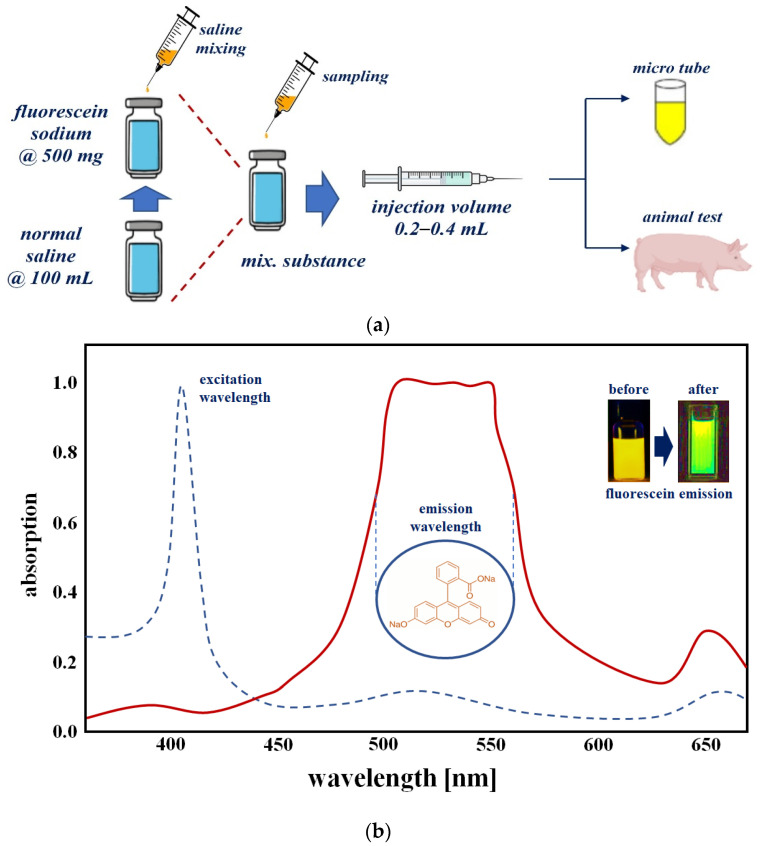
Phantom reagent preparation procedure (**a**) making for phantom (**b**) absorption spectrum of fluorescein sodium.

A fluorescence contrast agent was used to obtain the experimental results for specular reflection removal images using phantom and animal experiments. The fluorescence contrast medium was fluorescein sodium, as shown in Figure 13a, and the fluorescein sodium (500 mg) reagent was prepared with the optimal concentration (5.0 mg/mL) of fluorescein sodium by dilution with normal saline (100 mL). Additionally, fluorescein sodium (500 mg/mL) was diluted with 100 mL of normal saline to ensure a dilution value of five times or more. Fluorescein sodium that satisfied the dilution produced an injection volume of 0.2–0.4 mL using a syringe. The fluorescein sodium (dilution) was injected (syringe: 0.2–0.4 mL) into the phantom, micro tube, and vein (for pre/clinical test).

Figure 13b shows the measurement results for absorption (for emission of fluorescein sodium) spectrum of a vial phantom (see Figure 13a) using the spectrometer (Thorlabs OSA201C). From the figure, the measured result of the wavelength and absorption level were from 510 nm to 560 nm and 0.97 with an irradiate wavelength of 405 nm. This can be changed to a green color using external optical irradiation. Therefore, the yellow color was changed to a green color. The wavelength of the green color is 500 nm to 600 nm.

**Figure 14:**
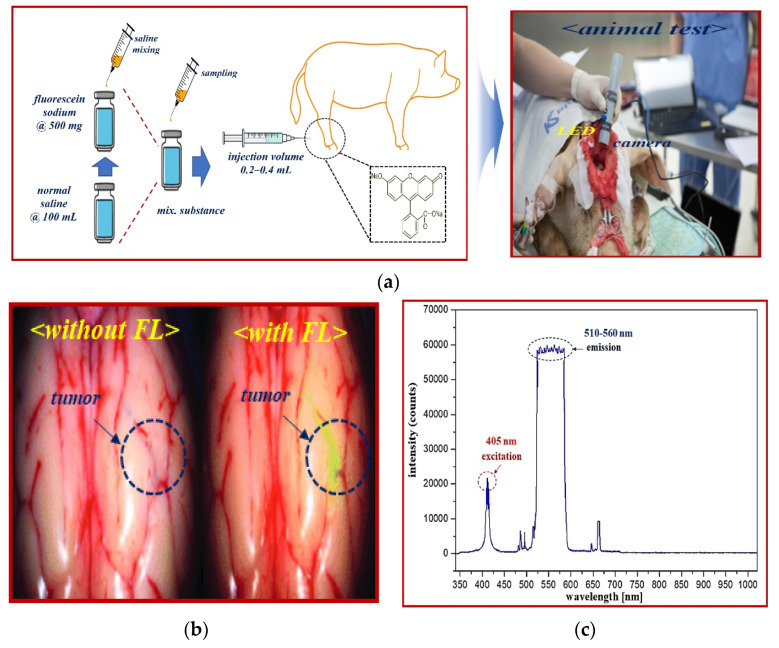
Fluorescein sodium mechanism of action and emission wavelength band. (**a**) Fluorescence expression animal test (**b**) Fluorescence detection after injection. (**c**) The excitation and emission wavelength of fluorescein sodium.

The experimental results obtained using vials were verified on animals for reliability testing. Large animals (pigs) were tested at the Animal center of Osong Medical Innovation Foundation (KBIO) after obtaining Animal Institutional Review Board (IRB) approval from the Animal Ethics Commission (KBIO-IACUC-2019-021). A typical male (farm) pig weighing 60 kg was used in this study. Fluorescein sodium (yellow dye) fluorescence contrast medium (concentration: 0.002 mg/kg) was injected intravenously (0.2–0.4 mL), as shown in Figure 14. Fluorescein sodium injected into a vein was combined with plasma protein, and fluorescein sodium circulated through blood vessels.

Fluorescein sodium with an injection volume of 0.2–0.4 mL was irradiated with an external light source such as an LED or laser. Fluorescein sodium has an excitation wavelength of 405 nm and has a characteristic of emitting fluorescence with a wavelength of 510–560 nm. Therefore, when an external light source of 405 nm such as LED is irradiated to the fluorescein sodium, a fluorescence of 510–560 nm is emitted. When an image was acquired using a NIR camera suitable for the emission wavelength, the vein (shown in green) was adapted to the fluorescence expression wavelength band, and eventually, the blood flow phenomenon of the NIR band was observed in the fluorescence expression-based blood vessel [48].

### 4.3. Experiment Device Configuration

**Figure 15:**
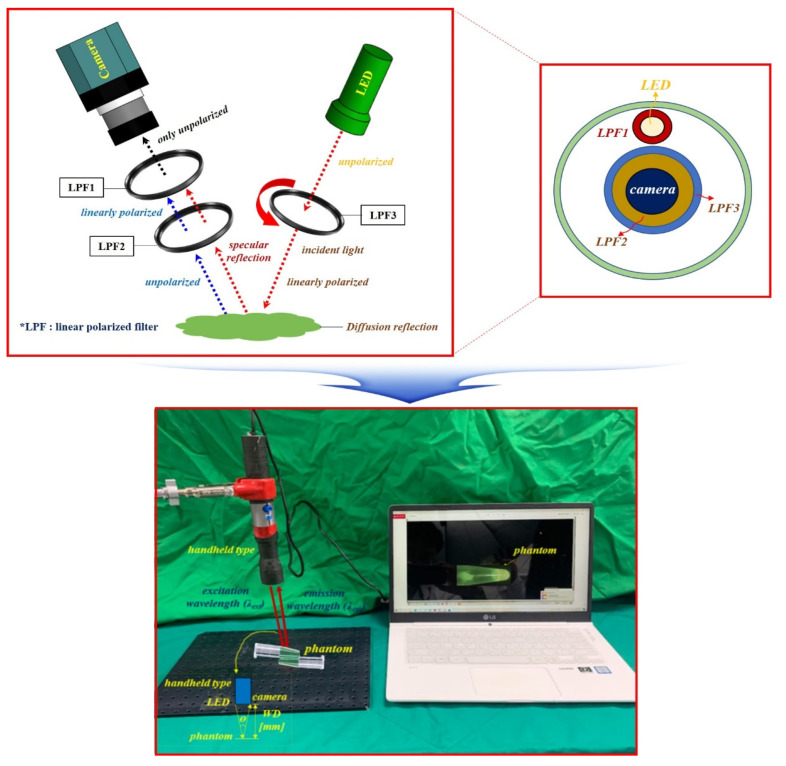
Experimental device set up.

To capture the effect of reducing specular reflection, the experimental apparatus was configured as shown in Figure 15. The experimental setup consisted of a camera, a linear polarized filter (LPF), and an LED. The results were obtained using a self-made fluorescein sodium mixing vial. The result of removing specular reflection was obtained by imaging and observation using a self-made handheld-type fluorescence surgical microscope. The handheld-type fluorescence surgical microscope had the following characteristics. The component devices included an LED, IR camera, LED light intensity control function, ON/OFF switch, power supply, and external monitor.

**Figure 16:**
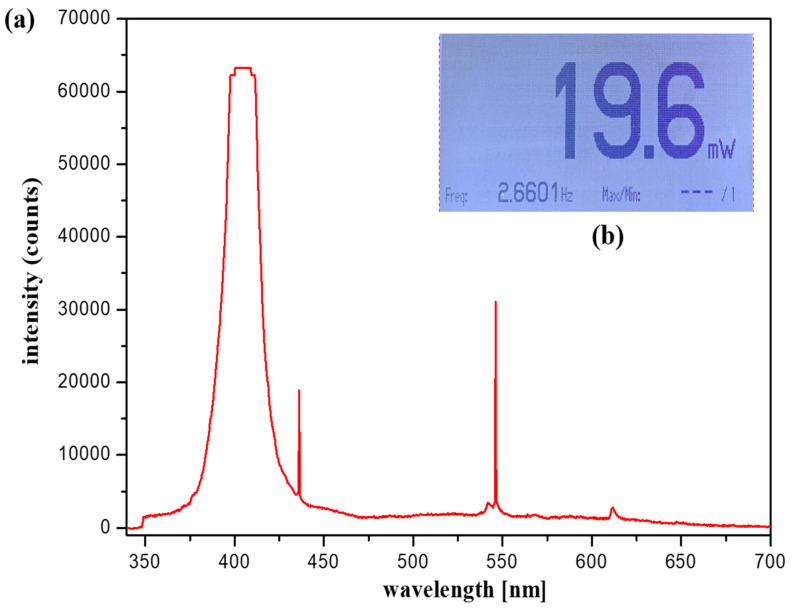
Light-emitting diode wavelength and output level (**a**) output level (**b**) output wavelength.

For accurate imaging, a self-made, handheld-type fluorescence surgical microscope was fixed in position using a clamp device. The working distance (WD) from the camera and the LED to the phantom was 3 cm, and the angle (*θ*) between the LED and the camera to focus the beam was approximately 15°. For the LED used in the experiment, the wavelength and output level of the light source were measured before use. Measurements were obtained using a spectrum meter and a power meter. As shown in Figure 16, the irradiation (λ_ext_) wavelength of the LED was 405 nm, and the irradiation power (P_ext_) was approximately 19.6 mW.

Table 4 shows the main parameters of the light emitting diode (LED) used in the experiment.

LPF_1_ was connected to the front end of the LED, and the rotation angle (*θ*) was 0°. In addition, a long pass filter (cut on wavelength: 500 nm @ thorlabs FEL0500) that could pass only the emission wavelength (510–560 nm) and two LPFs (LPF_1_//LPF_2_) were connected in front of the camera head. At this time, the rotation angle *θ* of the LPF_1_ was 0° (unpolarized), and the rotation angle *θ* of the LPF_2_ could be rotated from 0 to 360° (unpolarized or polarized). The method of adjusting the polarization state for specular reflection was essentially the regulation of the rotation angle (*θ*) of the LPF (F_2_) to 90° (polarized). The self-made handheld-type fluorescence surgical microscope was manufactured using 3D printer technology. The main parameters of the camera used for the experiment are presented in Table 5.

To utilize the linear polarized filter (Corning Polarcor™ Glass Polarizer), the wavelength band was 430–1100 nm. The diameter of the lens was 5.0 mm, and the thickness of the filter lens was 0.5 mm. The transmittance was 66%, the insertion loss was 1.8 dB, and the angle of the incidence beam was 5.0°.

## 5. Results and Discussion

**Figure 17:**
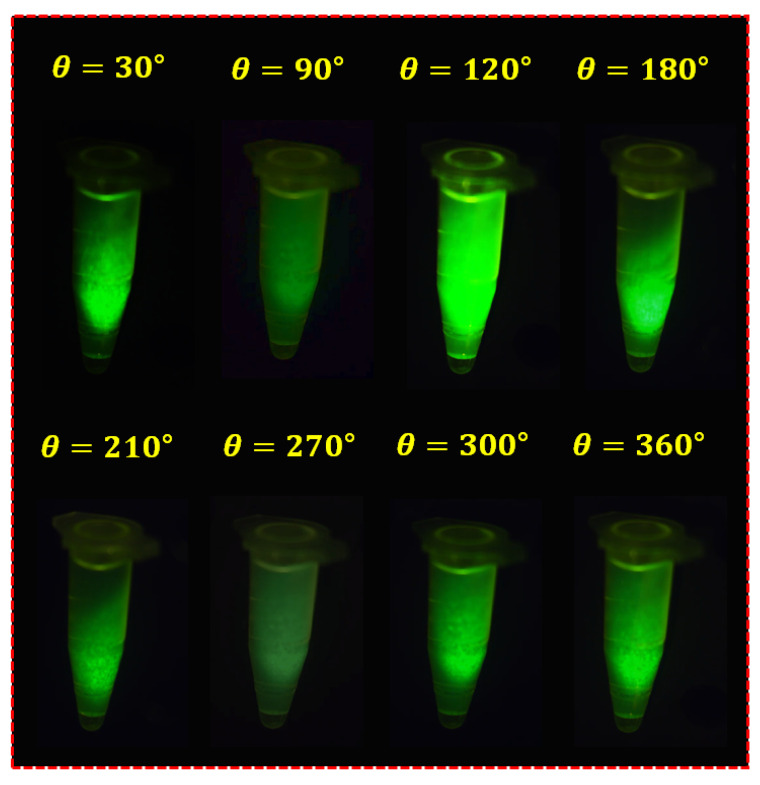
Result of fluorescein sodium vial phantom experiment with the LPF.

Figure 17 shows the results of the experiment based on the fabrication of the fluorescein sodium liquid vial phantom. The first filter (Filter_1_) was fixed, and the second filter (Filter_2_) was rotated at regular intervals to capture the image. When the rotation angle (*θ*) of the filter was rotated to 30°, 90°, 120°, 180°, 210°, 270°, 300°, and 360°, the image with reduced specular reflection could be observed. In particular, when the rotation angle (*θ*) was 90° and 270°, the specular reflection was sufficiently reduced, and other results were analyzed differently depending on the rotation angle (*θ*) in the intensity of the specular reflected light. To obtain reliable results, this experiment was performed five times (without LPF and with LPF states), and consistent results were obtained.

**Figure 18:**
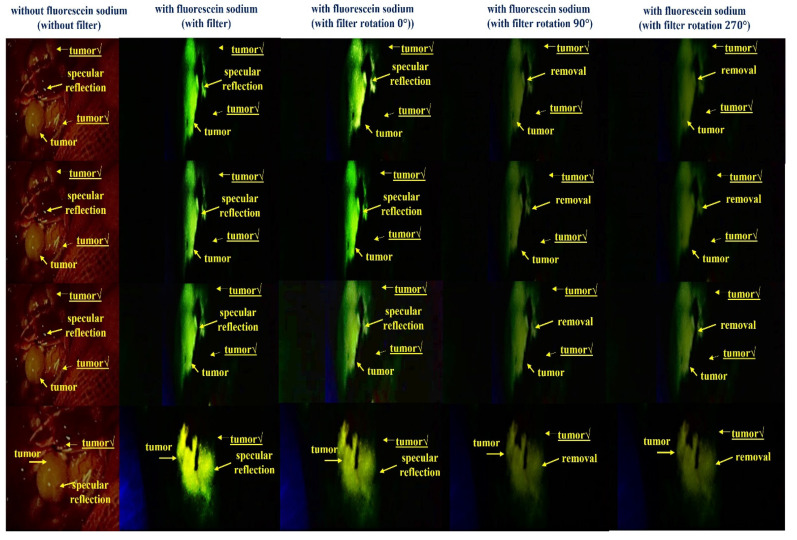
Result of the specular reflection removal experiment using large-animal experiment.

Figure 18 shows the results of a reflex removal experiment using a large animal (pig). As observed in the figure, the experimental results show the state of blood flow through the fluorescent sodium fluorescein. For fluorescence images, an LED mounted on a self-manufactured handheld fluorescence surgical microscope irradiated the picture, and image data were collected through NIR camera imaging. After that, the results of the imaging experiment could be observed through an external monitor. In addition, Figure 18 shows the entire tumor before fluorescein sodium injection in the without filter (without fluorescein sodium) section. At this time, it was confirmed that light reflection occurred, and the with filter (with fluorescein sodium) part comprised the same tumor (←tumor maker) as the without filter part, and the same part was photographed three times for reproducibility. At this time, in a state in which the tumor was partially removed, fluorescein sodium was injected, and fluorescence was stained. Therefore, it is the result of comparing and observing the fluorescence state (←tumor maker) when the tumor is present (←tumor) and the non-fluorescent state (←tumor√) when the tumor is removed. Therefore, the reason that the shape of the tumor in the without filter (without fluorescein sodium) and with filter (with fluorescein sodium) parts is inevitably different is that the difference between the state in which the tumor is not removed and the state in which the tumor is partially removed for the fluorescence expression test cannot be compared. When irradiated with a light source from the outside, the performance of the state in which fluorescence is not expressed in the removed tumor (←tumor√) and the state of fluorescence in the non-removed tumor (←tumor) was tested. At this time, it was confirmed that light reflection still occurred in the fluorescence-expressed tumor (←tumor), and because we used a filter to remove the light reflection, it was confirmed that the light reflection was sufficiently removed when three shots were taken.

As a result of adjusting the rotation angle (*θ*) of the filter from 0° to 360°, when the rotation angles were 90° and 270°, a fluorescence image in which reflection was sufficiently removed was confirmed. Therefore, the same results as the experiments using the phantom were confirmed through animal experiments.

The fluorescein sodium used in the experiment has the property of having a 2-benzofurans compound that is dependent on the xanthene dye and is converted and circulated into various derivatives. Tumors have a fluorescence wavelength of 505–530 nm and a fluorescence expression phenomenon due to the occurrence of derivatives for fluorescein sodium [49]. In addition, the 405 nm LED used in this paper directly irradiates the tumor with a low power of less than 20 mW. Therefore, the 405 nm LED used in the medical field has no adverse effect on surrounding tissues due to its low power. For this reason, 405 nm LED is widely used in the medical field [50,51]. In particular, the visible light LED with wavelengths of 400 to 700 nm is widely used in the medical field, as they have been found to have excellent sterilization effects and to be harmless to the human body [52]. In particular, the use of visible light LED is recommended for fluorescent contrast agents such as fluorescence sodium to avoid adverse effects on human tissues [53].

## 6. Conclusions

The main aim of surgery is to completely excise tumors. Since the tumor is mixed with normal tissues and blood vessels, complete resection of only the tumor is difficult. Therefore, a fluorescence imaging device is used to differentiate between the tumor and the vascular boundary. In the LED used for fluorescence imaging, some light is refracted by Snell’s law, and some light is reflected and photographed by the camera. The photographed specular reflection image may interfere with the observation field of an important lesion during the diagnosis process, which may cause a diagnosis error. In clinical diagnosis sites, most of the cameras adjust the orientation angle or the brightness of the LED to reduce specular reflections from the captured images. However, when the camera direction angle is adjusted, the lesion in the captured image may be distorted, or the shape observation angle may be changed. In addition, if the LED brightness is adjusted, the captured image is observed on a dark background. Therefore, the shooting result is corrected using image processing, which is time-consuming and inconvenient. A clear image for rapid and accurate diagnosis must be provided, and specular reflection must be removed to secure the observation field of the lesion. This study develops a method to effectively reduce the specular reflection of an image generated by a fluorescence imaging device, and this experiment effectively controls polarization based on the rotation angle of the filter. Specular reflection for vertical polarization is removed by adjusting the direction of the vertical polarization and horizontal polarization. Thereafter, the filter allows only the horizontally polarized light to pass through. This study enables securing the imaging results for the specular reflection removal effect using the phantom, and large animals are tested to ensure high reliability of the experimental results of the phantom and fluorescein sodium liquid. When comparing the results of the large animal test with the phantom and fluorescein sodium liquid test results, it was possible to confirm the reduction in the occurrence of specular reflection. The proposed method can remove specular reflections in real time in the operating room without additional software processing and correction processing. The proposed method can be integrated with all cameras, microscopes, and endoscopes, regardless of the size of the filter. In addition, such a method provides an excellent photographed image to the operator. Therefore, the lesion observation field can be secured through clear imaging results in the experiment without specular reflection, and accurate diagnosis results can be rapidly obtained. Therefore, this method can be applied to clinical diagnostic sites in the future, and this method is highly practical. The proposed method can be applied in the medical field through product design and clinical trials in the future.

## Figures and Tables

**Table 1:** Types of fluorescence imaging diagnostics.

Configuration	KINEVO 900 (Germany)	Spy Elite (Canada)	Flare (USA)	PDE (Japan)	Leica FL800 (Germany)
Fluorescein contrast agent	Yellow dye	ICG	ICG	ICG	ICG, 5-ALA
Excitation wavelength	560–590 nm	806 nm	670 nm	760 nm	820–860 nm
Working distance (WD)	62 cm	30 cm	45 cm	20 cm	47 cm
Food and Drug Administration	Approved	Approved	Approved	Approved	Approved

**Table 2:** Change of current intensity according to the rotation angle.

F_2_ Filter Rotation Angle (*θ*_r_)	Specular Reflection Intensity	F_2_ Rotation Angle of the Filter (*θ*_r_)	Specular Reflection Intensity
0°	$\theta_{r}=cos^{-1}\sqrt{\frac{2I_{EP}}{I_{ref}}}$	120°	$\theta_{r}=cos^{-1}\sqrt{\frac{I_{EP}}{I_{ref}}}$
30°	$\theta_{r}=cos^{-1}\left(\frac{2I_{EP}}{I_{ref}}\right)$	180°	$\theta_{r}=cos^{-1}\sqrt{\frac{I_{REF}}{I_{EP}}}$
90°	$\theta_{r}=cos^{-1}\left(\frac{I_{EP}}{I_{ref}}\right)$	210°	$\theta_{r}=cos^{-1}\sqrt{\frac{I_{REF}}{I_{EP}}}$

**Table 3:** Analysis of changes in the specular reflection intensity according to the rotation angle of the filter (F_2_).

F_2_ filter rotation angle (*θ*) (°)	0	30	90	120	180	210	270	300	360
specular reflection intensity (mW/cm^2^)	50.0	37.5	0.00	12.5	50.0	37.5	0.00	12.5	50.0

**Table 4:** LED main parameters.

Parameter	Specification
Excitation wavelength, λ_ext_ (nm)	405
excitation power, P_ext_ (mW)	40
bias voltage (V)	<5.6
LD reverse voltage (V)	2.0
Bias current (mA)	100
Beam divergence (°)	16

**Table 5:** Experimental device module parameters.

Parameter	Specification
Model	405
Image sensor	40
Pixel size (mm)	<5.6
Resolution (Pixels)	2.0

## Data Availability

The data presented in this study are available upon request from the corresponding author. The data are not publicly available because of privacy and ethical restrictions.
